# State-of-the-art surgical treatment of IPMNs

**DOI:** 10.1007/s00423-021-02349-9

**Published:** 2021-11-04

**Authors:** Roberto Salvia, Anna Burelli, Giampaolo Perri, Giovanni Marchegiani

**Affiliations:** 1 Department of General and Pancreatic Surgery, Verona University Hospital, Piazzale Scuro 10, 37134 Verona, Italy; 2 Department of Surgery, Dentistry, Paediatrics and Gynaecology, Verona University Hospital (Policlinico G.B. Rossi), Piazzale Scuro 10, 37134 Verona, Italy

**Keywords:** Intraductal papillary mucinous neoplasms, IPMN, Pancreatic ductal adenocarcinoma, Pancreatic cystic neoplasms, Pancreatic surgery

## Abstract

**Background:**

A “pandemic” of incidentally discovered pancreatic cyst neoplasms (PCNs) is ongoing. Among PCNs, intraductal papillary mucinous cystic neoplasms (IPMNs) are the most common and with their complex biology could represent a precursor lesion of pancreatic cancer. Although multiple guidelines exist to guide their treatment, there are still many “gray areas” on indications for surgery for IPMNs.

**Methods:**

The current indications for surgery of IPMNs were reappraised, considering potential discrepancies between available evidence and guidelines policies. The practice at a high-volume center for the diagnosis and treatment of PCN was presented and discussed.

**Results:**

Most IPMNs do not and will never require surgery, as they won’t progress to malignancy. The current literature is solid in identifying high-grade dysplasia (HGD) as the right and timely target for IPMN resection, but how to precisely assess its presence remains controversial and guidelines lack of accuracy in this regard. Multiple tumorigenic pathways of progression of IPMNs exist, and their knowledge will likely lead to more accurate tests for malignancy prediction in the future.

**Conclusions:**

The surgical management of IPMNs still is a matter of debate. Indication for resection should be considered only in highly selected cases with the ideal target of HGD. Clinicians should critically interpret the guidelines’ indications, refer to a multidisciplinary team discussion, and always consider the outcome of an adequate counselling with the patient.

## Overview

In the context of a modern “pandemic” of incidentally discovered pancreatic cyst neoplasms (PCNs) [1], intraductal papillary mucinous cystic neoplasms (IPMNs) are attracting increasing interest among the medical community, as they represent an entity so common and yet so difficult to frame inside a unique, straightforward clinical approach. Given that IPMNs represent a precursor lesion of pancreatic cancer [2], their treatment was originally based on a blind, aggressive surgical approach due to lack of reliable data on their biology and a high presumed risk of malignancy.

However, surveillance for selected cases has gained more and more favor thanks to the progressive availability of new evidence from large observational studies, highlighting how most cases can be safely surveilled over time due to a low risk of malignant progression [3–6].

Three main guidelines on the management of PCNs exist: (1) the guidelines of the International Association of Pancreatology (IAP), published in 2006 and updated in 2012 and 2016; (2) the European evidence-based guidelines published in 2013 and updated in 2017; and (3) the guidelines of the American Gastroenterological Association (AGA) published in 2015 [7–9]. These guidelines tend to be consistent regarding the main indications for surgery, while they diverge on the indications for surveillance and surveillance discontinuation. Despite their undoubtful clinical utility, guidelines are obviously mainly based on expert opinions. Recommendations are based on scientific evidence provided by few observational studies and mostly surgical series, which still probably overestimate the risk of developing a malignant disease [7–9]. This surgical bias explains why current guidelines are characterized by high sensitivity and low specificity and consequently burdened by high rate of surgical overtreatment. Large observational series were recently published aiming to describe the natural history of IPMNs, but lack of pathological confirmation was inevitably associated with risk of misdiagnosis and of underestimating malignancy [3, 5]. As a matter of fact, forty years after their identification, the natural history of IPMNs remains substantially unraveled. Therefore, a standardized evidence-based clinical management is lacking.

The purpose of the current article is to critically review the surgical treatment of IPMNs, focusing on the indications for surgery and on the various existing controversies between guidelines, evidence, and clinical practice.

## Epidemiology and biology of IPMNs

In an era characterized by an extensive use of cross-sectional imaging, a recent meta-analysis estimated a pool prevalence of incidentally discovered PCNs of around 8%, with a 4.7% of cysts “likely to have mucinous nature” [1]. Among pancreatic cysts, IPMNs have a prevalence ranging from 20 to 80% and a potential to progress to cancer following the adenoma-to-carcinoma sequence [10]. Their prevalence and the risk of malignant progression increase with age, with no significant difference between genders and pancreatic location [11]. Two classifications can be applied to IPMNs: morphological and histological. The first identifies main duct IPMNs (MD-IPMNs), branch duct IPMNs, and mixed type IPMNs (MT-IPMNs). Surgical series report a higher rate of malignancy in case of MD- and MT-IPMN (60–92%) compared to BD-IPMNs (6–46%) [12, 13]. Concerning histology, IPMNs are graded as low-grade dysplasia (LGD) or high-grade dysplasia (HGD), based on the architectural and cytologic atypia of their lining epithelium, and they are also subtyped according to the differentiation of this epithelium, as intestinal, gastric, pancreatobiliary, and oncocytic. Also, invasive cancer (IC) arising from IPMNs are subtyped according to their cytological characteristics as tubular, colloid, and oncocytic [14, 15].

The morphological classification of IPMN is relevant in the preoperative setting to assess the risk of malignant progression and, subsequently, to select the most appropriate management. However, the treatment after resection is guided by the presence or absence of an invasive component regardless of morphology [14–16].

## Diagnosis

### Clinical presentation and symptoms

Once overtaking the bias of surgical series, most IPMNs are asymptomatic [1, 17]. Among symptomatic patients, the majority complaints about aspecific symptoms as diffuse abdominal pain, dyspepsia, and bloating that, probably, do not even correlate with the presence of IPMN. Only very few signs and symptoms can be directly related to the presence of IPMNs, namely, obstructive jaundice, recurrent acute pancreatitis, new-onset or worsening diabetes mellitus, and steatorrhea due to endocrine or exocrine insufficiency. Yet, only jaundice was found to be an independent predictor of HGD or IC [18].

### Cross-sectional imaging and endoscopic ultrasound

At diagnosis, it is crucial to distinguish a presumed IPMN from other PCNs and to correctly assess specific radiological features. Computed tomography (CT), magnetic resonance imaging (MRI), and contrast-enhanced endoscopic ultrasound (CE-EUS) are the most widespread diagnostic tools. MRI with magnetic resonance cholangiopancreatography (MRCP) has shown superior to CT in identifying specific type of PCNs, communication with the pancreatic duct system, mural nodules, and multifocal PCNs [19]. However, the accuracy of radiological imaging in identifying specific PCNs subtypes remains low [20], ranging from 39 to 44.7% for CT and from 39.5 to 50% for MRI [21]. Therefore, when features suspicious for degeneration appear, a combined approach with CE-EUS may be required. Clinical guidelines are not unanimous with regard to indications to perform CE-EUS whose use is explicitly advocated only by IAP and AGA guidelines [7, 9]. The diagnostic accuracy of CE-EUS with or without fine needle aspiration (FNA) ranges from 40 to 96%, and its addition to abdominal imaging significantly increases overall diagnostic accuracy for PCNs [22, 23]. Furthermore, CE-EUS represent the gold standard in identifying mural nodules, helping to distinguish benign and malignant cysts [24–26]. The true advantage of EUS is to perform FNA of cyst fluid and to use it for cytological, biochemical, or DNA analysis. Analysis of CEA level using a cutoff of 192–200 ng/ml and cystic fluid amylase level proved to be useful in differentiating mucinous from non-mucinous PCNs [27]. A recent meta-analysis showed low pancreatic cyst fluid glucose level to have significantly improved diagnostic accuracy compared with CEA alone for the diagnosis of mucinous versus non mucinous PCNs [28]. Eventually, cytological examination is a highly specific test for the detection of malignancy with a specificity that approaches 100%, but this technique is hampered by the low cellularity of pancreatic cyst fluid and high heterogeneity of IPMNs. As a matter of fact, a single IPMN has multiple locules, and sample of one locule may not represent the entire lesion. Therefore, the sensitivity of cytopathology varies widely from 25 to 88% [2].

## Indications for surgery: between evidence and guidelines

Most patients with pancreatic cancer are diagnosed at a late stage. Therefore, IPMNs with HGD, pancreatic intraepithelial neoplasia 3 (PanIN 3), and mucinous cystic neoplasia (MCN) with HGD represent a unique chance for a surgical cure as they are precursor lesions of pancreatic cancer [2, 29]. Whereas PanINs cannot be detected at traditional imaging, IPMNs and MCNs with HGD can be macroscopically identified and represent, according to the International Cancer of the Pancreas Screening Consortium, the ideal target for surgery to prevent pancreatic cancer [30]. Knowledge on biology and evolutionary path of IPMNs is increasing in recent years. Once a cyst becomes invasive, the estimated average window for its development from HGD is 3 years. These data by Noë et al. confirmed, once again, non-invasive IPMNs as potential origins of pancreatic cancer, providing opportunities for early detection and intervention [31].

### Clinical and radiological parameters of guidelines

While waiting for more reliable predictor of malignancy, indications for surgery according to current consensus guidelines are still based on clinical and radiological parameters. MPD dilatation ≥ 10 mm, presence of enhancing mural nodule, jaundice, and malignant cytology have been historically recognized as risk factors for HGD and IC [2, 18, 32, 33]. Therefore, both the European and the IAP guidelines acknowledge them as indications for immediate surgery, namely, high-risk stigmata (HRS) and absolute indications (AIs) for surgery, respectively [7, 8].

Enhancing mural nodules are the strongest predictor of either HGD or IC for all types of suspected IPMNs [34]. Despite no evidence exists about a dimensional cutoff related to mural nodules, the risk of malignancy appears to be directly proportional to the size, and, according to guidelines, diameter ≥ 5 mm represents a clear indication for surgery [34].

While surgical series continue to identify MPD dilation ≥ 10 mm, and even between 5 and 9 mm, as one of the best predictors of HGD and IC, reinforcing the policy of surgical resection, data on surveilled patients suggest that, in the absence of other features suspect for malignancy, MPD dilation alone (above all between 5 and 9 mm) is characterized by a considerable risk of misdiagnosis and possible overtreatment [33, 35–37]. For this reason, it appears safe to suggest surveillance in asymptomatic patients who have “worrisome” MPD dilation (5–9 mm) but lack other absolute or relative indications. In these cases, the risk of misdiagnosis with other pancreatic diseases is indeed relevant.

Cyst size has been historically recognized as a good indicator of malignancy risk. The IAP and European guidelines consider cyst size ≥ 3 cm and ≥ 4 cm, respectively, as a surgical indication. Of note, both these cutoffs were chosen arbitrarily. Cyst, as well as mural nodule, size, although not directly related to malignancy, might suggest for how long the cyst (or the mural nodule) has been growing and therefore give an esteem of the increase of the risk of malignancy. To provide a workaround, instead of cyst size alone, growth rate during follow-up might be considered a more accurate parameter when predicting the risk of progression, rather than cyst size alone at first observation [38].

In general, those listed as worrisome features (WFs) and relative indications (RIs) in the IAP and European guidelines, respectively, should not be considered strong standalone predictors of malignancy. Indeed, patients with WFs had a significantly better 5-year disease-specific survival compared with those with HRS (96% vs 60%, respectively) [35]. Of note, whereas in the European guidelines, one RI warrants surgical resection in patients without significant comorbidities, in the IAP guidelines, the presence of at least one WF warrants further diagnostic evaluation with EUS in order to better scale the risk of malignancy.

Both the IAP and the European guidelines differ from the AGA guidelines that recommend surgery in case of MPD ≥ 5 mm and presence of solid component, or cytology positive for malignancy [9]. Of note, the AGA guidelines suggests discontinuation of surveillance once the patient is no longer fit for surgery (as the other guidelines) or if there has been no change in the features of the cyst after 5 years, unlike any other guideline.

### Dynamic predictors and indications for surgery beyond the guidelines

Guidelines policies have revealed their inaccuracy in predicting the risk of malignancy. The rate of LGD in surgical series applying such policies remains high (≥ 50%) with a peak of 77% in a recent multicentric snapshot study from US [18, 33, 36, 39]. Given the complication rate of pancreatic surgery, it does not seem acceptable that about half of the patients who undergo pancreatic resection for IPMN are overtreated or, at least, operated too soon. To supersede the inaccuracy of the above cited risk predictors of malignancy, the Verona group conducted an observational study focused on IPMNs “crossing-over” from observation to surgery to identify further dynamic predictors of malignant degeneration. They found that in BD-IPMNs under surveillance, development of additional WFs or HRS was associated with the presence of HGD, while harboring a stable WF carried the lowest risk of malignant disease [40]. Recently, the Heidelberg group presented the largest surgical series on IPMNs ever published by a single referral center [41]. In this study, among 1439 patients, timing of resection was categorized according to final pathology: too early (LGD); too late (IC); and timely (intermediate and HGD). Only 34% and 88% of the “timely” and “too late” group had radiographic criteria of suspicion, respectively. Therefore, the authors advocated a cautious application of watch-and-wait policies. On the other side, large observation studies have recently identified subgroups of patients affected by BD-IPMNs with a low risk of malignant degeneration. Pergolini et al. found that small (< 1.5 cm) BD-IPMNs without WFs have a significant lower risk of malignant degeneration compared to larger cysts [3], while the Verona group found that patients over 65 years of age with a “Trivial” cyst, defined as BD-IPMN not developing WFs or HRS in first 5 years of diagnosis, harbor the same risk of malignancy as the age-matched general population [38]. These data are consistent with those of Lee et al. that estimated an incidence of IC in patients with BD-IPMNs without significant changes within the first 5 years of surveillance around 1% and suggest that, when making surveillance decision of BD-IPMN beyond 5 years, the risk of malignant degeneration should be weighed against patients’ overall mortality risk [42].

Despite the inaccuracy of the existing tools, promising results are emerging from integrating cyst fluid genetics and clinical information. The application of an approach based on selected clinical features, imaging characteristics, and cyst fluid genetic and biochemical markers proved to be able to spare surgery in more than half of the patients who underwent resection of their cysts [43]. Hopefully, a combined approach including micro-RNAs and glycoproteins altered expression analysis and DNA testing of the pancreatic cyst fluid might be the key to better select the right candidates for surgery [43, 44].

### Eligibility of the patient and patient’s will

In addition to clinical and radiographic indications, the surgical decision-making should be based on eligibility of the patient for surgery, namely, on age, life expectancy, frailty, overall health status, and comorbidities. Adequate counseling should be used to understand the patient’s will and motivation for surgery and, conversely, the psychological burden associated with a surveillance program. Indeed, patients under surveillance for presumed IPMN at low risk of malignancy may present the “Sword of Damocles” effect, namely, initial subclinical symptoms of somatization, depression, anxiety, sleep disorders, and a reduced perception of physical role functioning in comparison to patients who received surgery for the same condition [45]. A cautious approach is of uttermost importance to select only candidates with appropriate oncological targets (HGD or IC), physical condition, and adequate motivation for surgery, maximizing the benefits of surgical resection and avoiding the burden of unnecessary surgery.

**Fig. 1:**
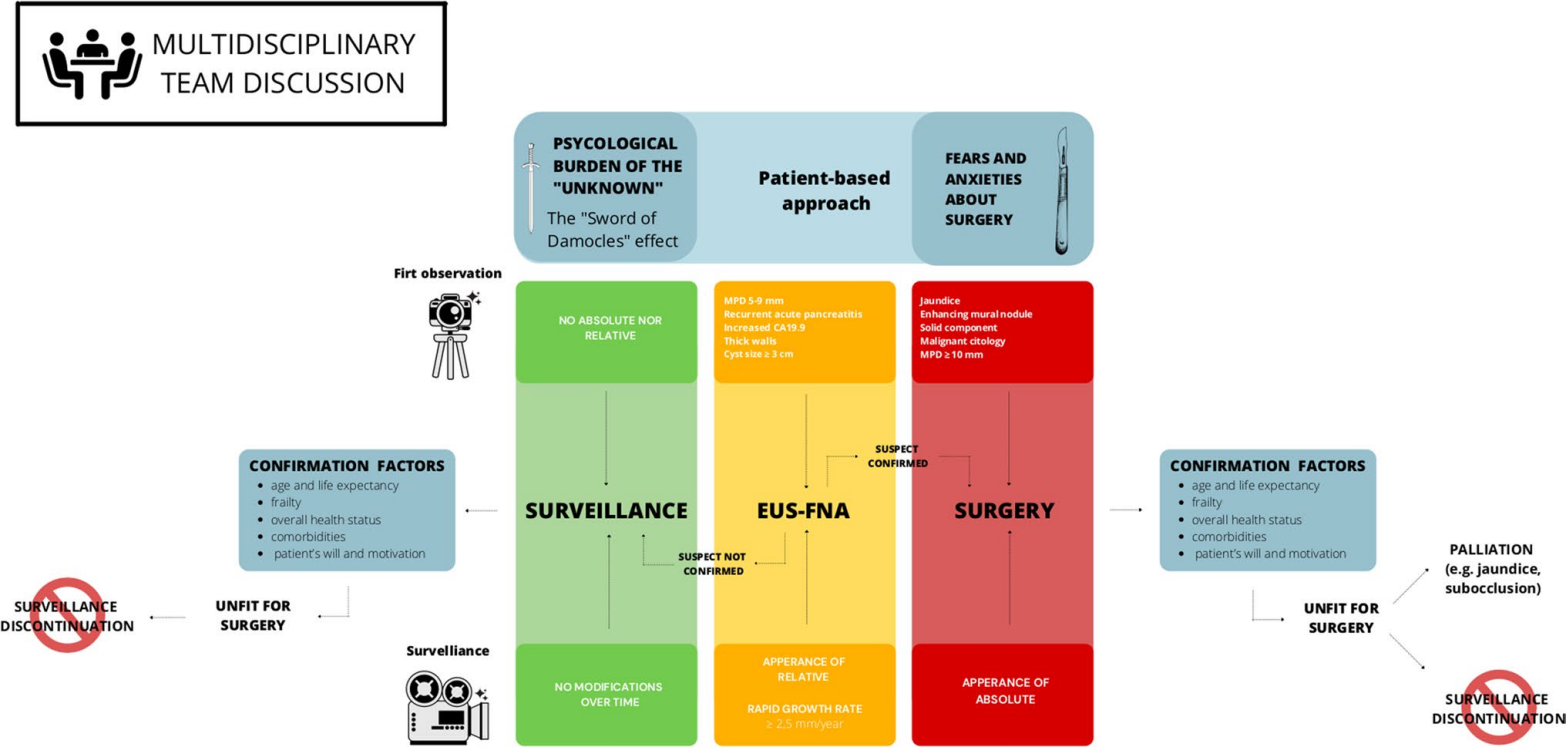
The clinical management of IPMNs at the Verona Pancreas Institute

Taking into account all these factors, the authors propose the algorithm displayed in Fig. 1 as the best current clinical management of patients diagnosed with an IPMN (Fig. 1).

## Surgery for IPMNS: intra- and postoperative implications

Given the complexity of diagnostic work-up for IPMNs and the experience needed to assure a safe pancreatic resection and associated postoperative course or, looking at the flip side of the coin, a “safe” surveillance, patients with a suspected IPMNs should be always referred to high-volume center and discussed in a multidisciplinary meeting before undergoing resection (Fig. 1). As the goal of surgery is the prevention of pancreatic cancer or its treatment in the earliest stage, oncological major pancreatic resections with standard lymphadenectomy are the goal standard [46]. According to the pancreatic location, pancreaticoduodenectomy (PD), distal pancreatectomy (DP), or total pancreatectomy (TP) should be performed, either with an open or minimally invasive approach. Even if several centers have experience in this regard [47, 48], parenchyma sparing non-oncological procedures such as middle pancreatectomy or enucleation should be avoided or limited to very selected cases [7, 8].

PD is indicated for segmental dilatation of the MPD at the pancreatic head or suspected lesions of the head, uncinate process, or neck of the pancreas. In general, major morbidity, defined as Clavien-Dindo ≥ 3, and mortality rates for PD are around 20% and 3%, respectively [49]. DP with or without splenectomy is indicated for lesion of the body and tail of the pancreas. Major morbidity and mortality rates range from 14% up to 38% and from 0% up to 2% after minimally invasive DP (MIDP) and open DP (OPD), respectively [50, 51]. TP is indicated in cases of diffuse MPD involvement, multifocal disease in patients with family history positive for PDAC, and persistent HGD at the resection margin because the established risk for recurrence and/or cancer in these patients makes the morbidity of TP more acceptable [52]. Moreover, recent studies have reported improved perioperative outcomes and postoperative quality of life (QoL) after TP, presumably due to centralization at high-volume centers and development of long-acting insulin and modern pancreatic enzyme preparations [53–57].

### Intraoperative frozen section

Intraoperative frozen section of the resection margin should always be performed during partial pancreatectomy in order to drive the extent of the resection [7, 8]. Whereas in case of HGD or IC further resection up to TP is needed, in case of LGD should be avoided [58]. Worryingly, in a recent survey on the application of guidelines, 49% of responders believed that an additional resection was required in case of LGD at frozen section [59]. One challenging finding in frozen section is the presence of ducts with denuded epithelium, especially if the duct is dilatated. Indeed, the presence of a denuded epithelium has been associated with recurrence [60]. In this case, the need of further resection should be evaluated carefully, considering the characteristics of the resected lesion, the presence or absence of other lesions in the remaining pancreas, and the clinical condition of the patient [15].

### Recurrence and progression after pancreatic resection

Resected invasive IPMNs recur in about half of the cases [61–65], while resected non-invasive IPMNs, namely, LGD or HGD, are associated with a risk of developing an IC in the pancreatic remnant around 2–3% [66–68]. Patients with resected non-invasive IPMNs have also a higher risk of developing additional features in the pancreatic remnant (e.g., development of a new cyst, increasing of MPD diameter, increasing of size of cysts in the pancreatic remnant) that is estimated around 20–25% [66–68]. Among all, increasing of MPD diameter after PD is the most challenging sign, given that in most cases it is a manifestation of a chronic obstructive pancreatitis rather than a sign of recurrence. These findings support the recommendation of the IAP and European guidelines that suggest lifelong follow-up regardless of tumor grade as long as the patients remains fit for surgery [7, 8]. Of note, the modality of follow-up after resection for IPMNs is not standardized and remains based on the experience of each center.

### Clonal heterogeneity and concomitant versus IPMN-derived pancreatic cancer

IPMN with “concomitant” carcinoma has been defined as the presence of two lesions separated by an uninvolved segment of pancreatic parenchyma [14, 15, 69]. However, this definition needs to be implemented, as it recently emerged that a genetically independent IC (namely, a concomitant IC) could arise also in the contest of an IPMN, making it indistinguishable from an IPMN-derived IC by clinical and pathological features alone [70]*.* These findings are consistent with the recent identification of different tumorigenic pathways and independent polyclonal origins for IPMNs that are superseding the traditional idea of the adenoma-to-carcinoma sequence as the only model for progression of IPMNs [71, 72]. Indeed, Omori et al. described three different pathways of IPMN progression. These are the sequential type, where all driver mutations are shared by PDAC and co-occurring IPMNs; the branch-off type, where some driver mutations are shared by PDAC and co-occurring IPMNs; and de novo type, where PDAC has driver mutations not shared with co-occurring IPMNs [71]. These results support the hypothesis of “field cancerization,” in which the entire pancreas of some patients is at increased risk for PDAC [73] and underlie, once again, how IPMNs are not an obligate precursor of PDAC. With this in mind, not only traditional clinical-radiological features appear unable to accurately predict the risk of malignancy, but also the most promising cyst fluid analysis, in case of PDAC adjacent to an IPMN, but genetically independent, would likely fail to identify mutations from high risk lesion, as the monitored cyst fluid would not contain any high-risk mutant DNA molecules of PDAC [70].

Of note, despite the huge clinical relevance that the understanding of the biology of IPMNs might have, a recent survey on the application of guidelines found that only 56% of responders usually distinguished between IPMN-derived IC and a concomitant PDAC [59].

## Open controversies

Although high quality evidence identifies HGD as the right and timely target for resection, the reality is much more complex. Many controversies and pitfalls still exist regarding surgical treatment of IPMNs:

1. IPMNs are genetically heterogeneous. Therefore, preoperative FNA or biopsy is usually not representative of the entire lesion. Moreover, not all low-grade lesions will progress to HGD and, eventually, to IC in an entire lifetime [71, 72].
2. IPMNs are often multifocal, and even unifocal non-invasive IPMNs are related to an increased risk of harboring malignancy in the pancreatic remnant [73]. Only TP can eliminate the risk of malignant progression, while partial pancreatectomies are inevitably related with an increased risk of progression, potentially even after resection of non-invasive IPMNs [66–68]. However, due to long-term postoperative outcomes, TP cannot be advocated for all IPMNs even for multifocal forms.
3. Radiological features, tumor markers, and clinical signs at present have proved to be inaccurate in predicting the risk of malignancy, setting the ground for the perception of surgical resection as the “best” treatment for IPMNs [41]. However, surveillance remains the option of choice for most IPMNs as the vast majority of “trivial” lesions will never progress [38]. Future research efforts should keep focusing on the right target for surveillance and even more on surveillance discontinuation.
4. Although combined approaches including DNA testing of the pancreatic cyst fluid are showing promising results in predicting the occurrence of IPMN-derived carcinomas, these cannot be effective in predicting the risk of a concomitant PDAC, both distant or adjacent to the IPMN [70].

## Conclusion

In conclusion, where and when to resect an IPMN remain a matter of debate. While waiting for more reliable tools, multidisciplinary team discussion, guidelines, and a patient-centered decision-making process should guide the treatment. The suggested clinical management of IPMN can be summarized as follows (Fig. 1). If no absolute nor relative indications for surgery are present at first visit or no modifications of the cyst appears over time, surveillance should be undertaken and proceeded, respectively. If relative indications are present at first visit or appears and/or growth rate is ≥ 2.5 mm/year over time, further diagnostic work-up with EUS-FNA should be performed. If absolute indications are present at first visit or appears over time, the patient should be advised for surgery. Every step of the clinical management should be discussed with the patient and confirmation factors as age and life expectancy, frailty, overall health status, comorbidities, patient’s will, and motivation must be evaluated. Once the patient is considered unfit for surgery for any reason, no further surveillance is required.

## Data Availability

Not applicable.
